# Gastroenterological complications in kidney transplant patients

**DOI:** 10.1515/med-2020-0130

**Published:** 2020-07-11

**Authors:** Armando Calogero, Monica Gallo, Antonello Sica, Gaia Peluso, Alessandro Scotti, Vincenzo Tammaro, Rosa Carrano, Stefano Federico, Ruggero Lionetti, Maurizio Amato, Nicola Carlomagno, Concetta Anna Dodaro, Caterina Sagnelli, Michele Santangelo

**Affiliations:** Department of Advanced Biomedical Sciences, University of Naples Federico II, Naples, Italy; Department of Molecular Medicine and Medical Biotechnology, University of Naples Federico II, Naples, Italy; Department of Precision Medicine, University of Campania Luigi Vanvitelli, Naples, Italy; Department of Public Health, University of Naples Federico II, Naples, Italy; Department of Clinical Medicine and Surgery, University Federico II, Naples, Italy; Department of Mental Health and Public Medicine, University of Campania Luigi Vanvitelli, Naples, Italy

**Keywords:** immunological and non-immunological complications, organ rejection, consequences of surgery, immunosuppressive therapy, infectious risk, development of neoplasms, gastrointestinal complications

## Abstract

Kidney transplantation is the surgical operation by which one of the two original kidneys is replaced with another healthy one donated by a compatible individual. In most cases, donors are recently deceased. There is the possibility of withdrawing a kidney from a consenting living subject. Usually, living donors are direct family members, but they could be volunteers completely unrelated to the recipient. A much-feared complication in case of kidney transplantation is the appearance of infections. These tend to arise due to immune-suppressor drugs administered as anti-rejection therapy. In this review, we describe the gastrointestinal complications that can occur in subjects undergoing renal transplantation associated with secondary pathogenic microorganisms or due to mechanical injury during surgery or to metabolic or organic toxicity correlated to anti-rejection therapy. Some of these complications may compromise the quality of life or pose a significant risk of mortality; fortunately, many of them can be prevented and treated without the stopping the immunosuppression, thus avoiding the patient being exposed to the risk of rejection episodes.

## Graphical abstract

**Figure:**
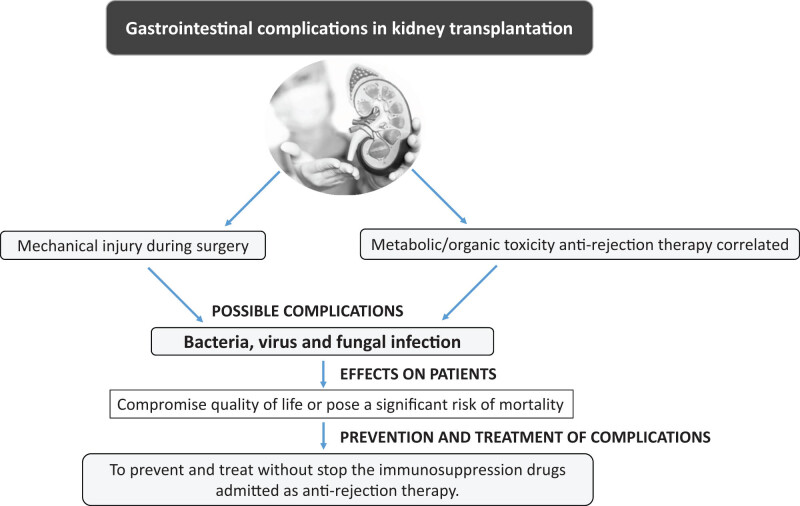

## 1 Introduction

Organ transplantation is one of the greatest achievements of modern medicine and allows a person who has lost his or her native organ’s functions to regain them and in some cases allow the survival of the patient [1]. The first kidney transplants in humans were made in the 50s and 60s of the last century and since then this practice evolved to become the best therapy for advanced renal failure. Transplantation is a surgical operation during which a kidney obtained from a donor (cadaver or living) is implanted inside the recipient’s body. In this case, kidney transplantation is a heterotopic transplant, that is, an organ is placed in a different location with respect to the native organs, which therefore are not removed (with the exception like polycystic kidney disease). The kidney is placed in the iliac fossa unless there are anatomical particularities that prevent it. The graft’s arteries and veins are joined to the recipient’s vessels and the ureter to the bladder, but this is not enough because for the transplantation to work a therapy is needed that allows the organism not to reject the transplanted organ. Currently, many drugs prevent acute kidney rejection with an act on the immune system of the transplanted patient; and if on the one hand they prevent the body from attacking the kidney, on the other hand, they lower the immune defenses, making it more susceptible to severe infections and tumors [2]. The duration of the transplant is variable (<5 or 5–20 years) and depends on several factors (renal disease, the organ transplanted characteristics, effectiveness of immunosuppressive therapy, etc.). After transplantation, the patient must take immunosuppressive therapy, until the kidney continues to function. Furthermore, the patient should undergo periodic checks, which must be very frequent in the first months after transplantation and can then be reduced over time. In the first months after transplantation, due to the powerful immunosuppressive therapy, the patient must limit contacts with people as much as possible. To evaluate the eligibility for transplantation, patients must undergo numerous physical and psychological tests. The age of the transplant candidate is a very important factor and even if there are no defined age limits, older patients may have more difficulty in being transplanted due to the presence of multiple pathologies. For older patients, it is possible to use transplants from older donors and therefore of compatible age.

 The condition of suitability for transplantation is subjected to continuous revision and immunological parameters are evaluated to identify the most suitable organ allocation to the patient s profile. Kidney transplantation in most cases is not a lifesaving transplant because dialysis allows survival of patients with renal failure. Compared to dialysis, transplantation is a therapeutic physiological alternative, especially in cases where the renal function returns to normal or close to normal. It allows people not to be dependent on dialysis and overcome the first months of isolation, so that they are able to move in full freedom and also re-enter fully into social and working life [3].

## 2 Types of renal transplantation

Kidney transplantation is the surgical procedure by which a kidney taken from a donor is placed in the body of a patient (recipient) with terminal renal insufficiency. There are several types of transplant, which can be classified according to the type of donor from which the organ originates.

### 2.1 Deceased donor kidney transplant

The organs destined to the donation are taken from patients whose death has been ascertained and with the consent or non-opposition of the family members. In order for an organ to be taken for a transplant, it is necessary that there are no serious diseases or conditions in the donor that could compromise the success of transplant or cause long-term complications for the recipient. Once the suitability of the donor has been ascertained, the organ is removed.

The following are the three types of cadaver donors:

(A) Organs come from donors whose brain death has been ascertained. Death is identified with the irreversible cessation of all brain functions due to primary or secondary encephalic damage (traumatic, hemorrhagic, and anoxic). This means that all the nerve functions that supervise life have ceased and that only the cardiorespiratory activity is artificially supported to guarantee the blood supply to the organs to be taken.
(B) Organs come from donors over 70 years old or over 60 years old with associated risk factors such as impaired renal function or presence of diseases (hypertension, diabetes, etc.). Once the suitability for the donation has been ascertained (renal tissue is evaluated), these organs are usually used for a double transplantation in a single recipient (also elderly).
(C) Organs come from donors who died of cardiac death in which any attempt at resuscitation was useless. Death is identified with the irreversible cessation of all vital functions determined by cardiocirculatory arrest with consequent irreversible loss of all functions of the brain. Once the subject (consent or non-opposition of the family) becomes a donor, he or she is subjected to well-defined procedures designed to maintain the fitness of the organs for the purpose of transplantation.

### 2.2 Living donor kidney transplantation

This procedure requires that the organ to be transplanted comes from a subject still alive. The donor must be of legal age and may be a kinsman (family member), a non-consanguineous associate affective to the recipient or an unknown person. The act of donation is totally free, always revocable and without any kind of constraint/coercion (to be understood both physically and psychologically) toward the recipient. The risk of surgery for the donor is the same as any other surgery and it is now widely demonstrated that with a single kidney it is possible to lead a normal life. However, both the donor and the recipient must be aware of the limitations of transplant therapy and aware of all possible clinical complications for both possible personal consequences and psychological implications. For these reasons an explicit and informed consent must be expressed, one must undergo a psychological–psychiatric exam and further evaluation by a special commission is required as well as the clearance of the judicial authority. The living transplantation is beneficial due to the fact the evaluation process of the candidates can be followed so that both the donor and the recipient are in the best conditions of health and it is possible to program the surgery. Furthermore, by reducing the time between organ harvesting and transplantation, better results can be achieved in relation to functional recovery and organ survival. The kidney harvesting surgery is done using laparoscopy procedure, which consists of four holes in the abdomen through which the kidney is extracted. In the absence of complications, the length of hospital stay is 3–4 days. Particular situations may require the need to prepare specific therapeutic and surgical protocols; these may include cases of transplantation (patients with previous transplantation) and the so-called “hyperimmune” patients (who have antibodies circulating already before transplantation).

### 2.3 Non heart-beating donor (NHBD)

NHBD is a type of donation in which the donor suffered a cardiac death, unlike the best known and used donation from deceased donors following brain death, or donors to beating heart (HBD). The spread of this type of transplant has remained for a long time limited to few countries (Japan, Holland, Spain and England) The term “death due to cardiac arrest” refers to the death that occurs following the cessation of the body circulation for a period of time that determines the irreversible loss of all brain functions, which is ascertained by a doctor by detecting the absence of a heartbeat (asystole) with a 20-min electrocardiogram. The deceased donor for cardiac causes is suitable for the removal of all organs except the heart; the privileged organs are the ones most resistant to ischemic damage, in particular kidneys, liver, and pancreas. Cardiac arrest is the peculiarity but also the main criticality of this mode of donation. During the asystole condition, the kidney does not receive blood supply due to the interruption of the circulation, the refrigeration of the organ and the subsequent reperfusion after the transplant, causing a type of damage defined as ischemia–reperfusion that is potentially higher than that observed in the transplant from a standard donor. To minimize this damage, organ perfusion techniques are used before and after the explant. After the explants, the organs can be kept at 4°C in special storage solutions or positioned inside a dedicated machine that, depending on the model, allows the organ to be perfused in a pulsatile or continuous way with a specific preservation liquid. The latter is the storage method used in the case of NHBD donors (non-beating heart). It has been widely demonstrated that the use of this technique significantly reduces the risk of delay in post-transplant functional recovery (delayed graft function (DGF)), improving the survival of the organ as well as better storage, and it also allows an assessment of suitability before surgery. As in the case of HBDs, organs taken from NHBDs are also assessed on the basis of a series of criteria to establish their suitability before surgery; in order for this type of program to work optimally, the presence of a team is necessary, consisting of various specialized professionals trained to act in a very short time. The data collected so far show a greater number of cases in which there is a delay in functional recovery (DGF) for a prolonged ischemia time. However, the available data show that this event does not affect the long-term survival of the transplanted organ. NHBD is a valid alternative to heartbeat transplantation and an extra chance to receive an organ.

### 2.4 Living donor kidney transplantation

Living donor transplantation is the best alternative in case of terminal chronic renal failure [4]. In fact, it has many advantages compared to the deceased donor transplantation such as a better degree of donor–recipient compatibility, a shorter waiting time and a reduction in damage related to organ preservation. In USA and in Japan, the percentage of living donor donation is very high; in Italy, on the other hand, there is little information concerning the living transplant, which unfortunately represents only 10% of the transplants performed. The law states that the spontaneous nature of the donation must always be ascertained to exclude any form of trade. In addition, the potential donor must be competent, aged no more than 75–80 years and “healthy”. In fact, although the focus is often on restoring health to the recipient, an equally important goal is to make sure that the donor can maintain his or her state of health after the donation. However, the assessment of eligibility requires a clinical evaluation and specific laboratory and instrumental tests. The donor is subjected to cardiological, vascular, pneumological, gastroenterological and hemocoagulative evaluation in order to exclude risks related to surgery, to nephrological exam to exclude that the removal of a kidney does not negatively affect survival or residual renal function and to immunological tests that highlight the degree of compatibility between donor and recipient; in addition, the presence of neoplasms or infections, such as hepatitis B, hepatitis C or HIV infection, which can be transmitted to the recipient, must be excluded. The candidate is subjected to a psychological evaluation in order to investigate the motivations that lead him or her to donate, the knowledge of possible risk factors and the anxieties about the success of the transplant; a more thorough psychological evaluation is made in case of altruistic donation, that is, to an unknown person. The kidney withdrawal surgery takes the name of nephrectomy and can be done in two ways: the laparoscopic procedure which consists of making four holes in the abdomen through which the laparoscopic instruments are inserted and a small incision through which the kidney is extracted or the laparotomic mode which consists of opening of the abdomen and is a little more invasive. In the absence of complications, the length of hospital stay is 3–4 days. The main risks of donation are related to surgery. The mortality of the donor linked to perioperative cardiovascular complications is however very modest and corresponds to an incidence of 0.03–0.05%, equaling to that reported in the most common surgical procedures. With regard to the long-term risks, careful selection of the donor allows the consequences to be very low; donors have a survival comparable to that of the general population. A fundamental advantage of living transplantation is related to time before the transplant; in fact, the longer you stay in dialysis, the lower the survival of patients after transplantation. The availability of a living transplant can reduce the stay in dialysis or even avoid it: in fact, a living donor transplant can be performed before the start of dialysis treatment (preemptive transplantation). The main obstacles to living donation are the presence of ABO blood group incompatibility between the donor and the recipient and the presence of antibodies in the recipient against the donor. In order to cope with these obstacles and therefore increase the possibility of donations from living donors, kidney transplantation can be performed between incompatible ABO subjects, treating the recipient with desensitization therapies about 1 month before and by plasmapheresis a few days earlier in order to eliminate antibodies against the donor. Since the organ demand is much higher than the supply of kidneys from a deceased donor, the living donor transplant represents a valid therapeutic option; i.e., being able to have a living donor allows a quick and safe transplantation without particular risks for the donor.

## 3 Complications related to kidney transplantation

Complications after a transplant can be related to the surgery itself (infection or abscess) or immunosuppressive therapy that the patient must continue for the life (cytomegalovirus [CMV] or viral infections) [5,6,7,8]. Several authors also reported in this setting of patients the risk of developing neoplasms like lung carcinoma and renal cell carcinoma [2,9] and lymphomas [10,11,12] (Table 1). Immunosuppressive therapy significantly reduces defenses against infections, so it is necessary to exclude any infectious or inflammatory condition before surgery. This therapy promotes the reduction in the body’s ability to control and eliminate cancer cells, so before receiving the organ it is necessary to exclude any condition of neoplasia or pre-cancerous disease in progress.

**Table 1:** The complications in kidney transplantation

	Etiology
Systemic infection	*Candida* spp., *Cryptococcus neoformans*, *Pneumocystis carinii*, cytomegalovirus
Gastrointestinal complications:	
*Esophagus–gastric infections*	CMV, Herpes simplex virus (HSV) and *C. albicans, C. krusei*, *C. tropicalis*, *C. parapsilosis*, *Torulopsis glabrata*
*Bacterial infection*	*Helicobacter pylori*, *Clostridium difficile*, *Campylobacter jejuni*, *Salmonella* spp.
*Viral infections*	CMV, HSV, Epstein-Barr virus.
*Diarrheal episodes*	Bacterial, viral and parasitic (*Caliciviruses* spp., HSV, *Clostridium difficile*, *Salmonella* spp., *Campylobacter jejuni*, and CMV)
*Immunosuppressive drugs*	Gastroduodenal ulcer and gastroduodenitis
Complications related to kidney transplantation:	
*Early complications*	Viral infections, abscess, colonic ischemia, pseudomembranous colitis, hemorrhages and infections related to treatment of acute rejection
*Complications after 1 year post-transplantation*	Diverticular disease, intestinal occlusion, colitis or neoplasms and intestinal perforation
Transplantation and risk of cancer	Lung carcinoma, renal cell carcinoma, lymphomas and leukemia

In particular, gastrointestinal complications can occur in many kidney transplant recipients. Such complications may be secondary to pathogenic microorganisms but may also be induced by mechanical or metabolic causes or by immunosuppressive drugs. Although this type of complication can compromise the quality of life of the transplant or be responsible for mortality, it can generally be prevented or treated without interfering with immunosuppression. Gastrointestinal complications are among the major causes of morbidity and mortality in immunosuppressed subjects [13,14,15] (Table 1). The digestive sphere represents, in fact, one of the privileged targets of clinical manifestations in patients with congenital immunodeficiency, in those subjected to organ transplantation or bone marrow or to immunosuppressive therapy for neoplasm or autoimmune disease [16,17]. This is may be due to the lymphoid tissue (macrophages and lymphocytic cells) localized in the chorion of the digestive mucosa. The onset of gastrointestinal infections in these subjects depends on the time from transplantation, age, the presence of chronic viral infections and pathologies present before transplantation (diverticular disease of the colon, peptic ulcer disease, diabetes, etc.), on the use prolonged of certain techniques (nasogastric tube), on exposure to pathogenic germs in community or hospital and on immunosuppressive therapy [18,19,20,21,22,23,24]. Budiño et al. [24] found that the main causes of death in 156 transplant recipients subjected to autopsy are infections, with a percentage ranging from 21 in heart transplant patients to 63 in those receiving a lung transplant [25]. Regarding the temporal appearance of gastrointestinal complications, in the first 30 days after transplantation, candida esophagitis or herpes simplex virus (HSV) and *Clostridium difficile* (CD) infection represent the most common pathologies. From 1 to 6 months after transplantation, viral and opportunistic infections occur, mainly from CMV; while after 6 months, 80% of patients are susceptible to infections for pathogens acquired in the community [26].

## 4 Esophagus–gastric infections

The esophagus is the organ most frequently affected by gastrointestinal complications in subjects receiving immunosuppressive therapy. The use of different prophylactic strategies, especially in the early phase of post-transplantation, has dramatically decreased esophageal and gastric infections. The most commonly implicated pathogens are CMV, HSV and *Candida* [27,28,29,30,31,32,33,34] (Table 1). Clinically, the most frequent symptom is dysphagia. Other modes of presentation of esophago-gastric lesions are the following: painful sensation during swallowing (odinophagia), retrosternal pain, dyspepsia, oral ulceration, and epigastralgia. In other studies, transplanted patients underwent endoscopy, with the identification of opportunistic infections in 64% of subjects with dysphagia, 39% with dyspepsia and 17% with hemorrhage; less common symptoms reported are nausea, vomiting, anorexia, hiccups and obstruction [35,36]. Most of these infections occur 2–6 months after transplantation, with a prevalence of 5% in kidney transplant recipients [37]. Candidiasis is the most common opportunistic infection in transplant patients, and *Cryptococcus neoformans* and *Pneumocystis carinii* can also be observed. Predisposing factors for infection are neoplasms, especially lymphomas and leukemia, organ transplants, prolonged therapy with antibiotics or corticosteroids, malnutrition, diabetes, and immunosuppressive therapy. Clinically esophageal candidiasis is diagnosed because of dysphagia, as the lesion of the mucosa is little or not ulcerated and the other symptoms, above all the painful ones (odynophagia, retrosternal pains, or epigastralgia) are rare. Nausea and hiccups or even asymptomatic forms are also observed [38]. Complications are rare and consist of hemorrhage, pseudotumoral stenosis, perforation or fistulization in the bronchi or mediastinum, while distant dissemination is exceptional. Therefore, the importance of esophageal candidiasis is not related to complications but to the possible repercussions on nutrition, and in the majority of cases, subjects already defected. Endoscopy is the examination of choice to obtain a diagnosis of certainty (Table 1). It can be postponed when the symptoms are striking and there is a diagnosis of esophageal candidiasis. In these cases, the probability of a relapse is so high that ex-juvantibus therapy is justified without further investigation [38]. Typical endoscopic manifestations are white-yellowish membranes or plates scattered on the esophageal mucosa, sometimes confluent, which can form longitudinal striae with a track-like appearance. The mucosa can be completely covered by pseudomembrane; and in some cases, the lumen can be obstructed. Membranes can rarely be blackish, while they are often easily detachable from the underlying mucosa, which may have a normal, erythematous, erosive or hemorrhagic appearance. In the case of descending candidiasis, i.e., starting from the oral cavity, the upper part of the esophagus is most frequently affected. The endoscopic aspect is so typical that biopsies are not indispensable and because of these lesions, differential diagnosis is not a problem. In uncertain cases, especially when the lesions are minimal, the diagnosis of certainty is evidenced by yeasts and filaments of mycetes, or by the cultivation of fungi from the biopsy samples. The culture test must be carried out when there is resistance to antifungal therapy in order to characterize the species of candida involved and provide an antifungigram. In fact, although *Candida albicans* is the most common species, infections are also caused by *C. krusei*, *C. tropicalis*, *C. parapsilosis*, *Torulopsis glabrata*, etc. (Table 1). Systemic antifungal therapy is used. Symptomatological improvement is usually rapid in 3–5 days. The duration of treatment ranges from 10 to 15 days [39]. The prophylaxis of fungal infections is different depending on the programs of the different transplant centers [40].

## 5 Viral infections

CMV infection is the most frequent, but other viruses such as HSV and Epstein–Barr virus can be due to serious esophagogastric complications [41] (Table 1). *Helicobacter pylori* (*H. pylori*) plays an important role in the pathogenesis of antral gastritis and peptic ulceration in the general population. *H. pylori* infection can be diagnosed by invasive methods, requiring endoscopy and biopsy sampling (histological examination, rapid urease test, culture, polymerase chain reaction) and non-invasive techniques such as urea breath test (breath test), the search for bacterium antigens on fecal samples or the search for specific antibodies in the blood and urine [42,43,44,45,46,47]. (Table 1). Among the non-invasive tests, only the breath test or the search for antigens of the bacterium in the stool allow to identify the infection in place and that is why they are called direct. The search for specific antibodies both in the blood and in the urine, on the contrary, represents only an index of successful exposure to *H. pylori* (immunological memory) but not of infection in progress and therefore are defined indirect tests. The prevalence of *H. pylori* is reported 70% in hemodialysed subjects and 60% in those undergoing kidney transplantation. Gastritis was found in 65% of kidney transplant patients and only in 19% of hemodialysis patients, suggesting that other factors contribute to the onset of gastric lesions [41]. Teenan et al. [40] performed endoscopy in 33 patients, 2–4 months after kidney transplantation, and identified 16 cases of duodenitis, 10 of gastritis and 4 of gastric ulcer. *H. pylori* was detected in 48% of cases of antrum gastritis; no association was observed between *H. pylori* infection and plasma levels of cyclosporine or corticosteroids [40].

## 6 Cytomegalovirus infections

CMV infections are mostly esophagitis. Unlike HSV, CMV can cause systemic symptoms such as fever and weight loss. Being an immunomodulatory virus, other pathogens such as Aspergillus may cause disease [48] (Table 1). CMV seems to facilitate the onset of bacterial, fungal and viral infections, through a direct immunosuppressive effect. The lesions can arise due to a reactivation of the latent virus or a primary infection. The reactivation of CMV occurs 1–4 months after organ transplantation. Immunosuppressive anti-rejection therapy can prolong this period of high susceptibility to infection; only rapamycin is associated with a low incidence of CMV infections [49]. Symptoms that are often painful are represented, in order of frequency, by odynophagia, retrosternal pains, and dysphagia. Endoscopy is essential for diagnosis [50], more frequently the lesions are located in the lower half of the esophagus (Table 1). The most typical aspect is that of ulcerations excavated in irregular margins, sometimes covered by fibrin, rarely hemorrhagic; it is also possible to observe only an erythema of mucosa, or an erythema with erosions and some pseudotumoral forms have been observed too. Deep biopsies must be performed on the bottom of the crater and on the margins (the viral particles are localized in the chorion) to highlight the characteristic intranuclear inclusions in the connective cells. If inclusions are not visible, one should systematically perform an immunohistochemical study associated with standard methods for detecting viral particles. The therapy is based on virostatic agents to be used in environments specialized for their toxicity. At the gastric level, CMV is found more rarely and manifests itself with epigastralgias [49,51]. The diagnosis is made with endoscopy showing the typical appearance of ulcerative gastritis, of unique or numerous mold ulcerations, or with the less typical appearance of erythematous gastritis. CMV inclusions are also present in biopsy samples of healthy mucosa, so sometimes the clinician faces the problem, not indifferent considering the toxicity of effective drugs, of deciding whether to treat asymptomatic subjects.

CMV is usually localized in the colon, both segmentally and widespread. Ileal lesions that determine a very serious necrotic enterocolitis have also been described. Diarrhea is associated with abdominal pain, while systemic symptoms such as fever are rare. Gastrointestinal hemorrhages, perforations and toxic megacolon are possible in patients with preexisting gastroenterological diseases. The virus can proliferate in vascular endothelial cells causing vasculitis, thrombotic phenomena and local ulcerations. When the damage is very extensive, an ischemic colitis may occur, which may be an early complication in the first 4 months after kidney transplantation [52]. The most frequently involved segments are terminal ileum, cecum and ascending colon up to hepatic flexure [53]. Colitis from CMV occurs in transplanted patients with isolated, wide and molded ulcerations prevailing in the right colon, and the clinical picture is often characterized by hemorrhagic manifestations. CMV infection usually occurs in the first 6 months after transplantation [54,55]. Diagnosis of infection is made with colonoscopy with deep biopsy samples that show typical nuclear inclusions [50]. The virostatic drugs are intravenously effective but do not protect against relapse [56]. CMV causes bloody diarrhea and ulcerative lesions in the colonic mucosa. They have been identified as pathogens of enteritis especially in transplant patients [25]. Nosocomial outbreaks are reported in patients undergoing solid organ transplants or with cancer [57].

## 7 Other gastrointestinal complications

Diarrhea is one of the most frequent post-transplant complications and may result in discontinuity of immunosuppressive therapy. Usually supported by bacterial, viral and parasitic causes, the onset can also be determined by drugs used in the post-transplantation period. Caliciviruses can cause a prolonged diarrhea that resolves by reducing the dosage of immunosuppressive drugs. HSV is a rare cause of colitis in transplant recipients (Table 1). Colonoscopy can detect erythema, fragility of the mucosa, aphthous ulcers and necrotic ulcerative lesions. The defined diagnosis is based on the isolation of the virus after culturing the biopsy specimens [58].

The incidence of diarrheal episodes is 12.6%, with 41.5% of cases related to infectious episodes and 34% correlated to drugs [59]. The average period of onset of diarrhea was about 10 months after transplantation (2–12 years). Furthermore, while 12% of the episodes occurred in the first month, 22% were diagnosed around 1–6 months post-transplant and 66% in the late period (after 6 months) [59].

The CD is responsible for about 50% of diarrheal episodes associated with antibiotic treatment (Table 1). The mode of presentation varies from asymptomatic pictures, to febrile enterocolitis, to toxic megacolon. It is common in patients with transplantation and often occurs in semiepidemic or endemic form, with transmission from person to person and with spores that persist on the surfaces of the environments.

Another infectious cause of post-transplant diarrhea is *Salmonella* infection, which has a clinical syndrome characterized by febrile diarrhea, with or without polymorphonuclear leukocytes in the feces. Bacteremia is common in patients transplanted with salmonella gastroenteritis: approximately 20–30% compared to 3–4% of non-transplant recipients [22]. *Campylobacter jejuni* is the most commonly reported bacterial cause of foodborne infection which causes gastroenteritis and diarrhea. The epidemiology is similar to that of *Salmonella* and the clinical syndrome is characterized by febrile diarrhea with nausea and vomiting, and about 70–85% of patients present leukocytes and blood in the stool. CMV causes a febrile syndrome with diarrhea, which can be associated with bleeding, possible perforation, ischemic colitis and/or toxic megacolon [22,60]. In a study evaluating gastrointestinal complications in 580 kidney transplant patients, six cases of CMV colitis were detected, with death in two patients [27]. In addition to the infectious causes, diarrhea can also be correlated with some immunosuppressive drugs that are able to alter the transit time or interfere with the complex system of the intestinal flora [61,62,63].

## 8 Gastrointestinal perforations

Colorectal complications after renal transplantation can occur both early and after transplantation and are a direct consequence of the biological modifications produced by immunosuppressive therapy [64] (Table 1). Early complications such as colonic ischemia, pseudomembranous colitis, hemorrhages or infections may be related to treatment of acute rejection. Complications at a distance from transplantation include diverticular disease, intestinal occlusion, colitis or neoplasms. Diverticular disease is the most common source of gastrointestinal complications, with most cases occurring after 1 year post-transplantation but has also been reported in the early post-operative phase. In particular, diverticulitis complicated by intestinal perforations, abscesses, phlegm or fistulas has been reported in approximately 1.1% of kidney transplant patients [65]. An increase in the incidence of diverticular disease and intestinal perforation has also been reported, without a clear explanation, in patients undergoing kidney transplantation with polycystic kidney disease [66,67].

## 9 Transplantation and risk of cancer

Undergoing an organ transplant can save lives, but it can also increase the risk of cancer [68,69,70,71,72,73,74,75,76,77,78]. Among patients who have undergone a solid organ transplant, the risk of getting cancer is 50% [79] (Table 1). Other diseases, incorrect lifestyles and anti-rejection drugs, are among the main responsible. The incidence of 12.6% of colorectal neoplasms has been observed in kidney transplant patients. The risk factors identified are age at transplantation, pre-transplant splenectomy, a history of pre-transplantation neoplasia and cigarette smoking [80]. Vera et al. [81] showed a significant increase in colorectal neoplasms after liver transplantation in subjects with primary sclerosing cholangitis and ulcerative colitis. The risk factors for the development of colorectal neoplasia have been colonic dysplasia after transplantation (*p* < 0.00003), duration of colitis for more than 10 years (*p* < 0.002) and pancolitis (*p* < 0.0004). In these settings, a follow-up with a colonoscopy is recommended [81].

## 10 Immunosuppressive drugs and gastroenterological complications

As stated previously, complications after a transplant can be immunological and non-immunological (Table 1). The most important immunological complication of renal transplantation is the rejection that can be acute or chronic. To prevent or mitigate the recipient’s immunological response, it is subjected to a scheme of immunosuppressive therapy with the intake of one or more drugs that limit host defense responses, modulating one or more stages of rejection. The transplanted patient, subjected to immunosuppressive therapy, can present some specific complications. In fact, immune-suppressor drugs with their intrinsic toxicity are also responsible for multiple complications involving the digestive system. Sirolimus is an immunosuppressive drug used to prevent rejection in kidney transplants. The effect of sirolimus on causing oral ulcers is known [82,83]. Three cases of gastroduodenal ulcer bleeding are reported in patients receiving sirolimus [83]. The causes of ulcer in these patients are multifactorial and sirolimus has certainly played an important role in slowing the tissue-healing processes despite ongoing anti-secretory therapy. In the literature is reported a case of abdominal pain caused by gastroduodenitis from leukocytoclastic vasculitis in a kidney transplant patient on sirolimus. Both the symptomatology reported by the patient and the histological lesion on the antral level regressed after the suspension of the sirolimus [84].

## 11 Kidney transplantation and nutritional status

A concept that is emerging strongly in the field of clinical nutrition is the possibility of improving the course of a disease in different and serious conditions, thanks to the modulation of the diet. The state of nutrition can influence the trend, the prevalence of complications and the prognosis of many diseases and is variously compromised during illnesses. Dialysis patients are subject to profound metabolic and nutritional changes that modify the energy balance in a chronic and persistent form. Following the diet is essential for patients, regardless of the method adopted: the food plan makes the dialysis treatment more effective and improves the state of nutrition of the subject. Since the uremic condition is not perfectly correct by the dialysis methods, malnutrition in dialysis is present from 18% to 75% depending on the method used for the assessment of the state of nutrition and is one of the factors responsible for high mortality. On the other hand, also from this point of view, kidney transplantation represents the best substitution therapy for patients with chronic renal failure in terms of survival, quality of life and sensation of subjective well-being. Furthermore, transplantation represents the possibility of liberalizing the diet after the limitations imposed by conservative therapy and dialysis. However, most of the patients who have undergone renal transplantation tend to be overweight because the use of immunosuppressive and corticosteroids, generating among other side effects the increase in appetite, fat mass and retention of sodium and water. This increase will lead to higher calorie needs than the actual energy needs.

The outcome of kidney transplantation can be influenced by the patient’s nutritional status. Malnutrition, obesity and other metabolic complications can be prevented or corrected by appropriate food interventions. Weight control therefore plays a fundamental role in therapy and lifestyle. Furthermore, as described above, gastrointestinal complications can occur in many subjects undergoing transplantation. The first tool to be used is proper nutrition, supported by any nutritional approaches, to reduce the symptoms of gastroesophageal pathologies [14]. The diet to be followed will not be very different from that of an individual who is not transplanted and will be much easier to follow than that prescribed during dialysis. Currently, only a few randomized clinical trials on the best nutritional treatment after kidney transplantation are available. Furthermore, it is important to develop guidelines for optimizing the nutritional status of patients with chronic kidney disease at all stages and for the management of patients undergoing kidney transplantation. In the future, further studies should be performed to evaluate the effect of nutritional manipulation in kidney transplant patients and to provide data for a structured, multidisciplinary dietary approach to post-transplant nutritional care.

## 12 Conclusions

Organ transplantation represents one of the most important clinical achievements of the second millennium due to the positive clinical implications in the treatment of numerous unfavorable and not effectively treatable diseases. In recent years, several therapeutic protocols have been developed and perfected, which more specifically seek to inhibit rejection, trying to safeguard at least in part a residual capacity of the immune system to defend the organism with the reduction in the side effects related to the chronic use of drugs. Currently, the 5-year survival from kidney transplantation is more than 90% in transplant recipients. However, the anti-rejection immunosuppressive drugs necessary for the success of the transplant and for the patient’s survival are directly associated with an increased frequency of infections and tumors associated with viral infections, which are the main causes of morbidity and mortality in people undergoing kidney transplantation; in terms of incidence this risk is quantifiable by about 2- to 3-fold increase in developing any post-transplant *de novo* tumor. Prolonged exposure to immunosuppressive drugs, in fact, seems to negatively influence the ability of antiviral and antitumor immunosurveillance and to enhance the carcinogenic effect of some risk factors, such as ultraviolet rays and some drugs, that seems to promote carcinogenesis through mechanisms independent of the immunosuppressive mechanism. These evidences indicate the need to prepare appropriate screening/surveillance models in these patients to prevent or at least anticipate the diagnosis of post-transplant tumors. In this context, even the correct eating habits can represent an optimal secondary prevention strategy in reducing the risks of transplant rejection due to non-immunological reasons and morbidity and mortality from post-transplant complications.
